# Astaxanthin Inhibits Mitochondrial Dysfunction and Interleukin-8 Expression in *Helicobacter pylori*-Infected Gastric Epithelial Cells

**DOI:** 10.3390/nu10091320

**Published:** 2018-09-18

**Authors:** Suhn Hyung Kim, Joo Weon Lim, Hyeyoung Kim

**Affiliations:** Department of Food and Nutrition, Brain Korea 21 PLUS Project, College of Human Ecology, Yonsei University, Seoul 03722, Korea; cigdoli2@naver.com (S.H.K.); jwlim11@yonsei.ac.kr (J.W.L.)

**Keywords:** astaxanthin, *Helicobacter pylori*, mitochondrial dysfunction, gastric epithelial cells, reactive oxygen species

## Abstract

*Helicobacter pylori (H. pylori)* infection leads to gastric inflammation, peptic ulcer and gastric carcinoma. *H. pylori* activates NADPH oxidase and increases reactive oxygen species (ROS), which induce NF-κB activation and IL-8 expression in gastric epithelial cells. Dysfunctional mitochondria trigger inflammatory cytokine production. Peroxisome proliferator-activated receptors-γ (PPAR-γ) regulate inflammatory response. Astaxanthin is a powerful antioxidant that protects cells against oxidative stress. The present study was aimed at determining whether astaxanthin inhibits *H. pylori*-induced mitochondrial dysfunction, NF-κB activation, and IL-8 expression via PPAR-γ activation in gastric epithelial cells. Gastric epithelial AGS cells were treated with astaxanthin, NADPH oxidase inhibitor apocynin and PPAR-γ antagonist GW9662, and infected with *H. pylori.* As a result, *H. pylori* caused an increase in intracellular and mitochondrial ROS, NF-κB activation and IL-8 expression, but decreased mitochondrial membrane potential and ATP level. Astaxanthin inhibited *H. pylori*-induced alterations (increased ROS, mitochondrial dysfunction, NF-κB activation, and IL-8 expression). Astaxanthin activated PPAR-γ and its target gene catalase in *H. pylori*-infected cells. Apocynin reduced ROS and inhibited IL-8 expression while astaxanthin did not affect NADPH oxidase activity. Inhibitory effects of astaxanthin on ROS levels and IL-8 expression were suppressed by addition of GW9662. In conclusion, astaxanthin inhibits *H. pylori*-induced mitochondrial dysfunction and ROS-mediated IL-8 expression by activating PPAR-γ and catalase in gastric epithelial cells. Astaxanthin may be beneficial for preventing oxidative stress-mediated gastric inflammation-associated *H. pylori* infection.

## 1. Introduction

*Helicobacter pylori* (*H. pylori*) is a Gram-negative, microaerophilic bacterium that infects more than half of the world’s human population*. H. pylori* infection can cause chronic inflammation of the stomach, and result in gastritis, peptic ulcers, mucosa-associated lymphoid tissue lymphoma and gastric carcinoma [1]. In 1984, Marshall and Warren were the first to discover *H. pylori* in gastritis and peptic ulcer patients and to identify this pathogen as being causally associated with gastric diseases [2]. 

*H. pylori* stimulates cytokine release and activation of inflammatory mediators [3]. In particular, upregulation of the inflammatory chemokine interleukin-8 (IL-8) is pronounced in *H. pylori*-infected patients who manifest high levels of IL-8 in their gastric mucosa [4]. Increased IL-8 in gastric mucosa is associated with severe chronic gastritis and active progression of gastric cancer [5]. IL-8 recruits neutrophils to the site of infection and stimulates the generation of reactive oxygen species (ROS) [6]. Previous studies have revealed that *H. pylori* activates oxidant-sensitive transcription factors, such as the nuclear factor kappa-light-chain-enhancer of activated B cells (NF-κB), which induce IL-8 gene expression [5,7,8]. ROS function as signaling agents that mediate *H. pylori*–induced activation of NF-κB and subsequent IL-8 gene expression [9,10,11]. Nicotinamide adenine dinucleotide phosphate (NADPH) oxidase, a well-known source of ROS, constitutively produces superoxide anion and hydrogen peroxide [12]. *H. pylori* activates NADPH oxidase, leading to an increased production of ROS [13,14]. In addition, mitochondria, the center of energy production, are known to be the major source of ROS. Mitochondria produce superoxide anions as byproducts of the leakage of electrons from the mitochondrial respiratory chain [15]. 

Mitochondria are also known to be a target of oxidative damage. Uncontrolled overproduction of ROS can impair the mitochondria [16]. It is proposed that increased oxidative stress by NADPH oxidase causes mitochondrial dysfunction and subsequent increase in mitochondrial ROS production. A recent study demonstrated that ROS produced by NADPH oxidase can induce mitochondria dysfunction and ROS production [17]. Mitochondrial ROS can act as signaling agents to activate inflammatory signaling, induce expression of pro-inflammatory cytokines and stimulate inflammasome formation. [18,19,20]. However, the involvement of mitochondrial dysfunction and mitochondria ROS in *H. pylori*-induced gastric inflammation has not been studied.

Peroxisome proliferator-activated receptor gamma (PPAR-γ), originally implicated in adipocyte differentiation and regulation of lipid metabolism, has been recently noted for its anti-inflammatory activity. Studies showed that PPAR-γ activation can suppress inflammatory cytokines in macrophages and endothelial cells [21,22]. PPAR-γ regulates oxidative stress-induced inflammatory response by inducing the expression of antioxidant enzymes such as catalase [23,24]. Among the three PPAR subtypes, PPAR-γ is most prominently expressed in gastrointestinal epithelial cells [25,26,27]. Several studies have found that PPAR-γ agonists inhibit NF-κB activation and cell proliferation in gastrointestinal cells [26,28]. In addition, PPAR-γ alleviates inflammatory signaling by activating antioxidant genes such as catalase. However, the possible role of PPAR-γ in inflammatory cytokine expression in *H. pylori*-infected tissues has not been well studied.

Astaxanthin is a xanthophyll carotenoid that is abundant in algae, and in aquatic animals such as salmon, trout, krill, and lobster. Owing to the presence of a long conjugated carbon-carbon double bond backbone functionalized with polar hydroxyl and keto groups at each terminus, astaxanthin has a particularly strong antioxidative capacity compared to other carotenoids [29]. Numerous studies have demonstrated the anti-oxidative and anti-inflammatory effects of astaxanthin [30]. Some of these studies showed that astaxanthin exerts its anti-inflammatory effect in leukemia cells and macrophages by acting as a PPAR-γ agonist [31,32]. Based on these findings, we hypothesized that astaxanthin might suppress *H. pylori*-induced ROS generation and cytokine production through activation of PPAR-γ.

The aim of the study described below was to determine if *H. pylori* induces mitochondrial dysfunction and ROS-mediated IL-8 expression in gastric epithelial cells, and if astaxanthin inhibits *H. pylori*-induced mitochondrial dysfunction, NF-κB activation and IL-8 expression via PPAR-γ activation. To determine astaxanthin’s mechanism of action, the NADPH oxidase inhibitor apocynin, and PPAR-γ antagonist GW9662 were administered to *H. pylori*-infected cells alone or in combination with astaxanthin.

## 2. Materials and Methods 

### 2.1. Reagents

Astaxanthin was purchased from Sigma-Aldrich (St. Louis, MO, USA). Astaxanthin was dissolved in dimethyl sulfoxide (DMSO) and stored under nitrogen gas at −80 °C. Before treatment, the astaxanthin stock solution was thawed and added to fetal bovine serum to achieve the desired concentrations. NADPH oxidase inhibitor apocynin and PPAR-γ antagonist GW9662 were purchased from Sigma-Aldrich and dissolved in DMSO.

### 2.2. Cell Line and Culture Condition

The human gastric epithelial cell line AGS (gastric adenocarcinoma, ATCC CRL 1739, Rockville, MD, USA) was purchased from the American Type Culture Collection (Rockville, MD, USA). The cells were grown in complete medium consisting of RPMI 1640 medium (GIBCO, Grand Island, NY, USA) supplemented with 10% fetal bovine serum, 2 mM glutamine, 100 U/mL penicillin, and 100 μg/mL streptomycin (Sigma-Aldrich). The cells were cultured at 37 °C under a humidified atmosphere consisting of 95% air and 5% CO_2_.

### 2.3. Bacterial Strain and H. pylori Infection

The *H. pylori* strain NCTC 11637 was obtained from the American Type Culture Collection. The bacterial cells were grown on chocolate agar plates (Becton Dickinson Microbiology Systems, Cockeysvile, MD, USA) at 37 °C, under microaerophilic conditions, using an anaerobic chamber (BBL Campy Pouch^®^ System, Becton Dickinson Microbiology Systems, Franklin Lakes, NJ, USA). AGS cells were cultured and seeded overnight to reach 80% confluency. The *H. pylori* was harvested from chocolate agar plates, suspended in antibiotic-free RPMI 1640 medium supplemented with 10% fetal bovine serum, and then added to the AGS cell culture at a cellular ratio of 50:1. 

### 2.4. Experimental Protocol

To investigate the effect of astaxanthin, the AGS cells (1.0–1.5 × 10^5^/mL) were pre-treated with astaxanthin (1 or 5 μM) for 3 h before adding the *H. pylori*. Following 1 h of incubation the ROS levels, mitochondrial membrane potential, ATP level, IκBα level, NF-κB DNA binding activity, PPAR DNA binding activity, protein expression of PPAR-γ and catalase, and the activities of NADPH oxidase and catalase were determined. Following a 4 h incubation period, the IL-8 mRNA expression was measured, and following 24 h, the IL-8 level in the medium was assayed. To determine the involvement of NADPH oxidase, the cells were pre-treated with apocynin (0.2 or 1 μM), a NADPH oxidase inhibitor, for 3 h before exposure to the *H. pylori*. To ensure the involvement of PPAR-γ, the PPAR-γ antagonist GW9662 (5 μM) and astaxanthin (5 μM) were preincubated with the AGS cells for 3 h prior to the addition of the *H. pylori*

### 2.5. Preparation of Cell Extracts

The cells were harvested by treatment with trypsin/EDTA, followed by centrifugation at 1000× *g* for 5 min. The cell pellets were resuspended in lysis buffer containing 10 mM Tris pH 7.4, 15 mM NaCl, 1% NP-40 and protease inhibitor complex (Complete; Roche, Mannheim, Germany), and lysed by drawing the cells through a 1-mL syringe with several rapid strokes. The resulting mixture was incubated on ice for 30 min followed by centrifugation at 13,000× *g* for 15 min. The supernatants were collected and used as whole cell extracts. For the preparation of nuclear extracts, the cells were extracted in buffer containing 10 mM HEPES (pH 7.9), 10 mM KCl, 0.1mM EDTA, 1.5 mM MgCl_2_, 0.05% NP-40, 1 mM DTT, and 0.5 mM phenylmethylsulfonylfluoride (PMSF). The nuclear pellets were resuspended on ice in nuclear extraction buffer containing 20 mM HEPES (pH 7.9), 420 mM NaCl, 0.1 mM EDTA, 1.5 mM MgCl_2_, 25% glycerol, 1 mM DTT, and 0.5 mM PMSF and then centrifuged. The supernatants were used as nuclear extracts. To prepare the cytosolic and membrane extracts, the supernatants were separated by centrifugation at 100,000× *g* for 1 h. The membrane extracts were obtained by resuspending the pellets in lysis buffer containing 50 mM HEPES, pH 7.4, 150 mM NaCl, 1 mM EDTA, and 10% glycerol. The supernatants were used as the cytosolic extracts. The protein concentration was determined by using the Bradford assay (Bio-Rad Laboratories, Hercules, CA, USA).

### 2.6. Real-Time PCR Analysis for IL-8

Total RNA was isolated by using TRI reagent (Molecular Research Center, Inc., Cincinnati, OH, USA). Total RNA was converted into cDNA by reverse transcription through treatment with a random hexamer and MuLV reverse transcriptase (Promega, Madison, WI, USA) at 23 °C for 10 min, 37 °C for 60 min and 95 °C for 5 min. The cDNA was used for real-time PCR with specific primers for human IL-8 and β-actin. The sequences of the IL-8 primers used to produce the desired 297 bp PCR product are 5’-ATGACTTCCAAGCTGGCCGTGGCT-3’ (forward primer) and 5’-TCTCAGCCCTCTTCAAAAACTTCT-3’ (reverse primer). For β-actin cDNA production, the desired 349 bp PCR product was obtained by using the forward primer 5’-ACCAACTGGGACGACATGGAG-3’ and reverse primer 5’-GTGAGGATCTTCATGAGGTAGTC-3’. For PCR amplification, the cDNA was amplified by 45 repeat denaturation cycles at 95 °C for 30 s, annealing at 55 °C for 30 s, and extension at 72 °C for 30 s. During the first cycle, the 95 °C step was extended to 3 min. The β-actin gene was amplified in the same reaction to serve as the reference gene.

### 2.7. Enzyme-Linked Immunosorbent Assay (ELISA) for IL-8

The cells (1.5 × 10^5^/well) were seeded in 6-well plates. The supernatants were centrifuged at 15,000× *g* for 15 min at 4 °C and collected for measuring level of IL-8. The concentration of IL-8 in the medium was determined by using an ELISA kit (Biosource International, Inc., Camarillo, CA, USA).

### 2.8. Measurement of Intracellular and Mitochondrial ROS Levels

For the measurement of intracellular ROS, the cells were treated with 10 μg/mL of dichlorofluorescein diacetate (DCF-DA; Sigma-Aldrich) and incubated in 5% CO_2_/95% air at 37 °C for 30 min. DCF fluorescence was measured (excitation at 495 nm and emission at 535 nm) with a Victor5 multi-label counter (PerkinElmer Life and Analytical Sciences, Boston, MA, USA). For the measurement of mitochondrial ROS, the cells were treated with 10 μM MitoSOX red (Life technologies, Grand Island, NY, USA) and incubated in 5% CO_2_/95% air at 37 °C for 30 min. The MitoSOX fluorescence was measured (excitation at 514 nm and emission at 585 nm) using a Victor5 multi-label counter (PerkinElmer Life and Analytical Sciences, Boston, MA, USA). ROS levels were determined from the relative increases in fluorescence.

### 2.9. Measurement of Mitochondrial Membrane Potential (MMP) and ATP Level

To determine changes in MMP, the cells were first cultured on glass coverslips coated with poly-L-lysine, and then treated with astaxanthin, followed by treatment with *H. pylori*. Next, the cells were incubated with JC-1 reagent (*v*/*v* 1:100; 10009908, Cayman Chemical Company, Ann Arbor, MI, USA) for 20 min. After removing the media, the cells were dried for 15 min at room temperature and washed with PBS for 5 min, twice. The cells were mounted with mounting solution (M-7534, Sigma Aldrich). Red or green JC-1 fluorescence (excitation at 590 nm and emission at 610 nm and excitation at 485 nm and emission at 535 nm, respectively) were examined with a laser-scanning confocal microscope (LSM 880, Carl Zeiss Inc., Oberkochen, Germany). Fluorescent images were expressed as the percentage ratio of the red and green fluorescence densities using NIH Image J 5.0 software (National Institutes of Health, Bethesda, MD, USA). The average intensity per cell was determined, and more than 50 cells in each experimental group were analyzed.

The ATP level was quantified using a luminescent ATP detection assay kit (ab113849; Abcam, Cambridge Science Park, Cambridge, UK). The luminescent substrate solution was added to whole cell extracts and the luminescence was measured by using a microplate reader (Molecular Devices, Sunnyvale, CA, USA). The relative ATP concentration was determined from the ATP standard reference by interpolation. 

### 2.10. Electrophoretic Mobility Shift Assay (EMSA)

The NF-κB gel shift oligonucleotide (5’-ACTTGAGGGGACTTTCCCAGGGC-3’) and the PPAR gel shift oligonucleotide (5’-CAAAACTAGGTCAAAGCTCA-3’; sc-2587, Santa Cruz Biotechnology, Santa Cruz, CA, USA) were radiolabeled using [^32^P]-dATP (Amersham Biosciences, Piscataway, NJ, USA) and T4 polynucleotide kinase (GIBCO, Grand Island, NY, USA). The radiolabeled oligonucleotide was separated from unconsumed [^32^P]-dATP using a Bio-Rad purification column (Bio-Rad Laboratories) eluted with Tris-EDTA buffer. Nuclear extracts of the cells were incubated with the [^32^P]-labeled oligonucleotide in buffer containing 12% glycerol, 12 mM HEPES (pH 7.9), 1 mM EDTA, 1 mM DTT, 25 mM KCl, 5 mM MgCl_2_, 0.04 μg/mL poly[d(I-C)] at room temperature for 30 min. The samples were subjected to electrophoretic separation at 4 °C on a nondenaturing, 5% acrylamide gel. The gel was dried at 80 °C for 2 h after which it was exposed at −80 °C to a radiography film using intensifying screens.

### 2.11. Western Blot Analysis for IκBα, PPAR-γ, and Catalase

Aliquots from whole-cell extracts were loaded onto 8–10% SDS polyacrylamide gels (6–40 μg protein/lane) and separated by electrophoresis under reducing conditions. The proteins were transferred onto nitrocellulose membranes (Amersham, Inc., Arlington Heights, IL, USA) by electroblotting. The transfer of protein was verified using reversible staining with Ponceau S. The membranes were blocked using 3% non-fat dry milk in TBS-T (Tris-buffered saline and 0.2% Tween 20). The proteins were detected using antibodies for IκBα (sc-371, Santa Cruz Biotechnology, Dallas, TX, USA), PPAR-γ (sc-7273, Santa Cruz Biotechnology), catalase (ab16731, Abcam), and actin (sc-1615, Santa Cruz Biotechnology) in TBS-T solution containing 3% dry milk, and incubated overnight at 4 °C. After washing with TBS-T, the primary antibodies were detected using horseradish peroxidase-conjugated secondary antibodies (anti-mouse, anti-rabbit, anti-goat), and visualized by exposure to BioMax MR film (Kodak, Rochester, NY, USA) using the enhanced chemiluminescence detection system (Santa Cruz Biotechnology). Actin served as a loading control.

### 2.12. Determination of NADPH Oxidase and Catalase Activities

NADPH oxidase activity was measured by using a lucigenin-based assay. The assay was performed in 50 mM Tris–MES buffer, pH 7.0, containing 2 mM KCN, 10 μM lucigenin and 100 μM NADPH as the substrate. The reaction was started by the addition of membrane extracts containing 10 μg protein. The photon emission was measured every 15 s over a 5 min period using a microtiterplate luminometer (Micro-Lumat LB 96V luminometer, Berthold, NH, USA). NADPH oxidase activities present in cytosolic extracts were also monitored and used as negative controls. Catalase activity was measured using a catalase assay kit (ab83464; Abcam, Cambridge, UK). Changes in the H_2_O_2_ levels in whole cell extracts were measured colorimetrically and used to calculate the catalase activities defined in unit/mg protein

### 2.13. Statistical Analysis

One-way ANOVA, followed by Newman-Keul’s post hoc tests, was used for statistical analysis. All values in the results were expressed as the mean ± S.E. of three different experiments. A *p*-value of 0.05 or less was considered statistically significant.

## 3. Results

### 3.1. Astaxanthin Reduces ROS Levels and Inhibits Mitochondrial Dysfunction in H. pylori-Infected AGS Cells

**Figure 1:**
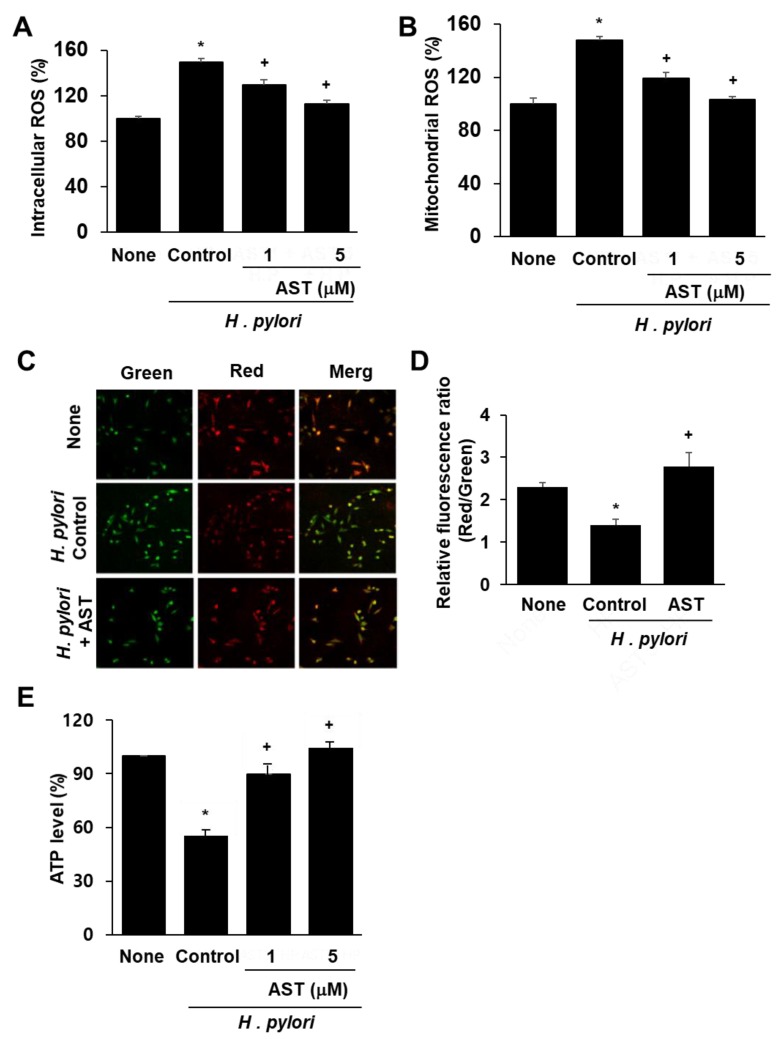
Effect of astaxanthin on ROS levels and mitochondrial dysfunction in *H. pylori*-infected AGS cells. The cells were pre-treated with the indicated concentrations of astaxanthin for 3 h and then stimulated with *H. pylori* for 1 h. (**A**) Intracellular ROS levels measured by DCF-DA fluorescence for uninfected AGS cells (None), AGS cells infected with *H. pylori* (Control; * *p* < 0.05 vs. None), AGS cells infected with *H. pylori* and treated with 1 or 5 μM astaxanthin (AST; + *p* < 0.05 vs. Control). (**B**) Mitochondrial ROS levels were measured by MitoSOX fluorescence and reported the same as in (**A**). (**C**) MMP of AGS cells without infection (None), infected with *H. pylori* alone (Control) or with *H. pylori* infection plus treatment of 5 μM astaxanthin (+AST) and stained with JC-1 dye and visualized with a confocal laser scanning microscope. (**D**) Relative ratios of red and green fluorescence densities of uninfected AGS cells (None), *H. pylori*-infected AGS cells (Control; * *p* < 0.05 vs. None) and *H. pylori* infection plus treatment of 5 μM astaxanthin (+AST; + *p* < 0.05 vs. Control). (**E**) Relative intracellular ATP levels measured for uninfected and infected AGS cells. See (**A**) for key to figure labels.

DCF-DA and MitoSOX fluorescence techniques were used to determine intracellular and mitochondrial ROS levels. Infection of AGS cells with *H. pylori* significantly increased the levels of intracellular and mitochondrial ROS (Figure 1A,B). Astaxanthin reduced both ROS levels in *H. pylori*-stimulated AGS cells in a dose-dependent manner. 

To determine whether *H. pylori* induces mitochondrial dysfunction in AGS cells, their MMPs and intracellular ATP levels were measured. MMP was measured by cytofluorimetric analysis using JC-1 fluorescent probe. The membrane potential-sensitive color shift is caused by the formation of red fluorescent J-aggregates. The green and red fluorescence images of the AGS cells are shown in Figure 1C. The ratios of green to red fluorescence by the AGS cells, reported in Figure 1D, reflect the relative cellular membrane potentials. *H. pylori* infection of AGS cells decreased the MMP as demonstrated by the observed decrease in the ratio of red/green fluorescence (see Figure 1D column ‘None” vs column “Control”). Astaxanthin (5 μM) prevented the *H. pylori*-induced decrease in the ratio of red/green fluorescence, which indicates that astaxanthin blocks *H. pylori*-induced decrease in AGS cell MMP (Figure 1D column “AST” vs. column “Control”). 

The data reported in Figure 1E show that the cellular ATP level decreased after *H. pylori* infection (column “None” vs column “Control”) and that pre-treatment with astaxanthin blocked this decrease (column “AST” vs column “Control”) in a dose-dependent manner. Because astaxanthin arrested *H. pylori*-induced decreases in the MMP and ATP levels, it is evident that it can prevent *H. pylori*-induced mitochondrial dysfunction in AGS cells.

### 3.2. Astaxanthin Inhibits NF-κB Activation and IL-8 Expression in H. pylori-Infected AGS Cells

**Figure 2:**
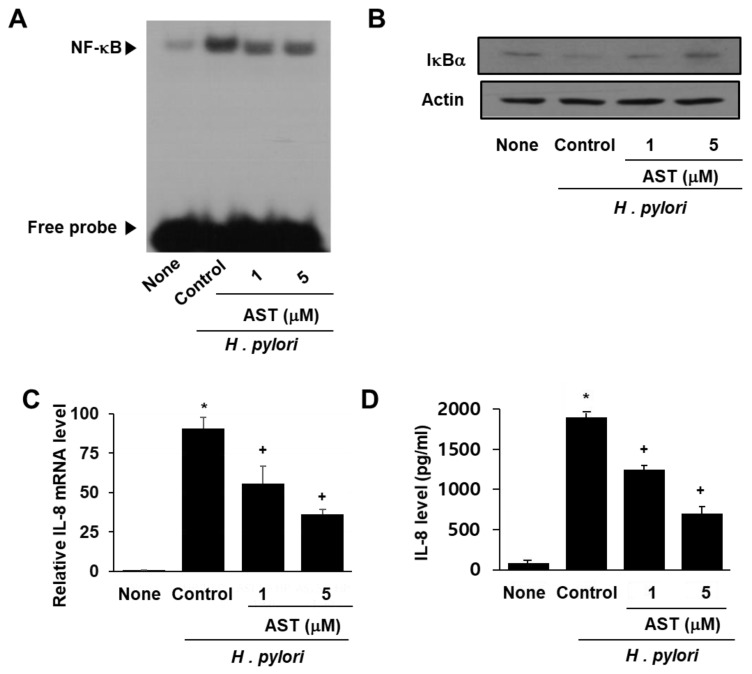
Effect of astaxanthin on NF-κB activation and IκBα and IL-8 levels in *H. pylori*-infected AGS cells. The AGS cells were pre-treated with the indicated concentrations of astaxanthin for 3 h and then stimulated with *H. pylori* for 1 h (for NF-kB activation, IκBα level), 4 h (for IL-8 mRNA level), and 24 h (for IL-8 protein level in the medium). (**A**) Audioradiogram of the EMSA gel on which nuclear extracts treated with a [^32^P]-oligonucleotide NF-κB probe were chromatographed. Column “None” corresponds to the uninfected AGS cell extract, column “Control” to the *H. pylori*–infected AGS cell extract, and columns “1” and “5”to the extracts of *H. pylori*–infected AGS cells pre-treated with 1 and 5 μM astaxanthin, respectively. (**B**) Western blot analysis of IκBα (and the protein standard actin) present in uninfected cells (column “None”), *H. pylori*–infected cells (column “Control”), and *H. pylori*–infected cells pre-treated with 1 and 5 μM astaxanthin, respectively (columns “1” and “5”). (**C**) Plot of the relative amounts of IL-8 mRNA present in uninfected AGS cells (column “None”), *H. pylori*–infected AGS cells (column “Control”), and *H. pylori*–infected cells pre-treated with 1 and 5 μM astaxanthin, respectively (columns “1” and “5”). (**D**) Plot of the concentration of the protein IL-8 in the media of uninfected AGS cells (column “None”), *H. pylori*–infected cells (column “Control”), and *H. pylori*–infected cells pre-treated with 1 and 5 μM astaxanthin, respectively (columns “1” and “5”). * *p* < 0.05 vs. “None”; + *p* < 0.05 vs. “Control”.

NF-κB is a protein complex that regulates the transcription of DNA in response to bacterial infection. Activation of NF-κB is inhibited by IκBα (nuclear factor of kappa light polypeptide gene enhancer in B-cells inhibitor, alpha). To demonstrate the effect of astaxanthin on NF-κB activation by *H. pylori*, the DNA binding activity of NF-κB and the level of inhibitory protein IκBα in infected AGS cells were determined by EMSA and Western blot analysis, respectively (see Figure 2A,B). *H. pylori* infection of the AGS cells resulted in increased NF-κB DNA binding activity compared to that observed for uninfected AGS cells (Figure 2A column “Control” vs. “None”). Pre-treatment of the AGS cells with astaxanthin prior to infection with *H. pylori* reduced the level of activity increase (Figure 2A columns AST “1” and “5”). 

Next, we examined the level of IκBα in AGS cells infected with *H. pylori*. IκBα is known to inhibit NF-κB formation and DNA binding activity. The data reported in Figure 2B indicate that infection by *H. pylori* decreases the level of IκBα in native AGS cells but not in the AGS cells pretreated with astaxanthin. Thus, astaxanthin prevents the decrease observed in IκBα induced by *H. pylori* infection.

Lastly, the impact of *H. pylori* infection on IL-8 mRNA and IL-8 protein levels was determined by using Real-time PCR and ELISA techniques, respectively. *H. pylori* increased cellular IL-8 mRNA as well as IL-8 protein concentration in the media (Figure 2C,D). Astaxanthin significantly reduced *H. pylori*-induced IL-8 mRNA and IL-8 protein in the cells in a dose-dependent manner.

### 3.3. NADPH Oxidase Inhibitor Apocynin Reduces ROS Levels and Inhibits IL-8 Gene Expression, but Astaxanthin Does Not Affect NADPH Oxidase in H. pylori-Infected AGS Cells

**Figure 3:**
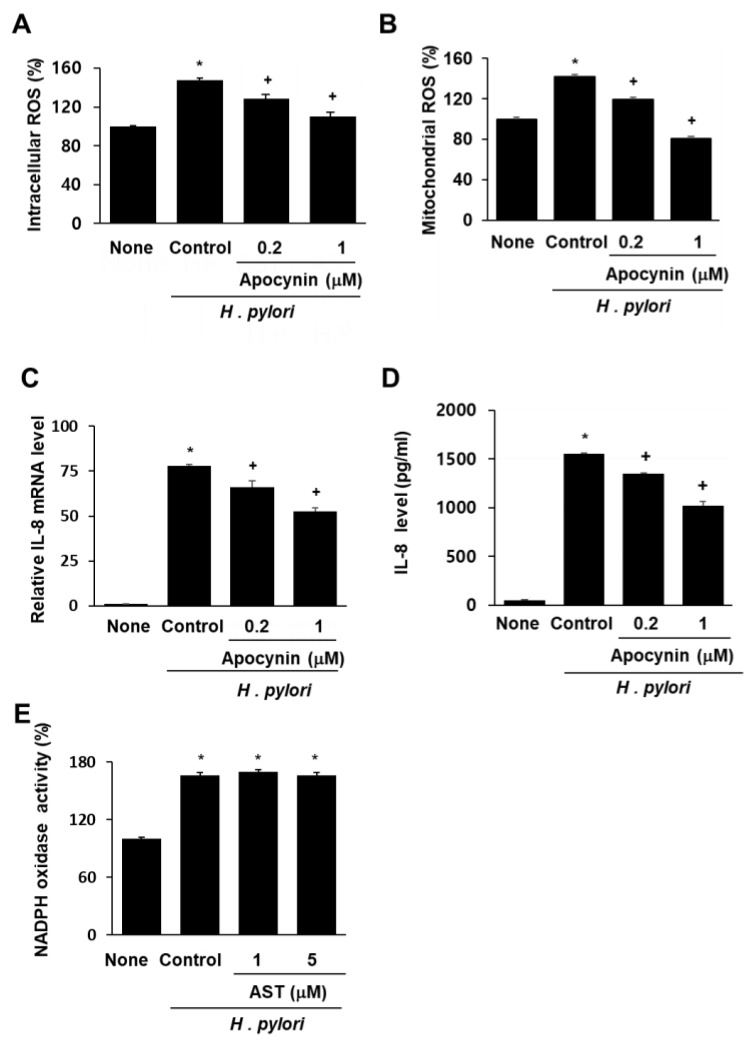
Effect of apocynin on ROS levels and IL-8 expression and effect of astaxanthin on NADPH oxidase activity in *H. pylori*-infected AGS cells. The AGS cells were pre-treated with the indicated concentrations of a NADPH oxidase inhibitor apocynin for 3 h and then stimulated with *H. pylori* for 1 h (for intracellular and mitochondrial ROS levels), 4 h (for IL-8 mRNA level), and 24 h (for IL-8 protein level in the medium). (**A**) Plot of the relative ROS level in AGS cells measured by DCF-DA fluorescence. Column “None” corresponds to uninfected AGS cells, column “Control” to *H. pylori*-infected AGS cells, and columns “0.2” and “1” to *H. pylori*-infected AGS cells pretreated with 0.2 and 1 μM apocynin, respectively. (**B**) Plot of the relative mitochondrial ROS level in AGS cells measured by MitoSOX fluorescence. The description of the columns is the same as in (**A**). (**C**) mRNA expression of IL-8 was determined by real-time PCR analysis. The description of the columns is the same as in (**A**). (**D**) Plot of the relative concentration of IL-8 in the media of cultured AGS cells determined by using the ELISA method. The description of the columns is the same as in (**A**). (**E**) Plot of the relative NADPH oxidase activity in AGS cells. The cells were pre-treated with 5 μM astaxanthin for 3 h and then stimulated with *H. pylori* for 1 h. NADPH oxidase activity was measured by lucigenin assay. The description of the columns is the same as in (**A**). * *p* < 0.05 vs. None; + *p* < 0.05 vs. Control.

To determine whether NADPH oxidase is involved in ROS generation and subsequent IL-8 gene expression in AGS cells infected by *H. pylori*, the impact of the specific NADPH oxidase inhibitor apocynin was determined. Pretreatment with apocynin decreased intracellular and mitochondrial ROS in AGS cells stimulated with *H. pylori* (Figure 3A,B). Similarly, *H. pylori*-induced IL-8 gene expression (determined at mRNA level and protein level in the medium) was inhibited by apocynin (Figure 3C,D). These results demonstrate that NADPH oxidase contributes to increases in both intracellular and mitochondrial ROS levels, and thus, to IL-8 gene expression in *H. pylori*-infected cells. To determine whether astaxanthin inhibits NADPH oxidase, the level of NADPH oxidase activity in *H. pylori*-infected cells pretreated with astaxanthin was measured and compared with that observed in untreated infected cells (Figure 3E). The *H. pylori* infection resulted in increased NADPH oxidase activity (column “Control” vs. “None”), and pretreatment with astaxanthin had no effect on this increase. These results show that the inhibitory effect of astaxanthin on ROS levels and IL-8 expression is not the result of NADPH oxidase inhibition.

### 3.4. Astaxanthin Induces Expression and Activation of PPAR-γ and Catalase in H. pylori—Infected AGS Cells

**Figure 4:**
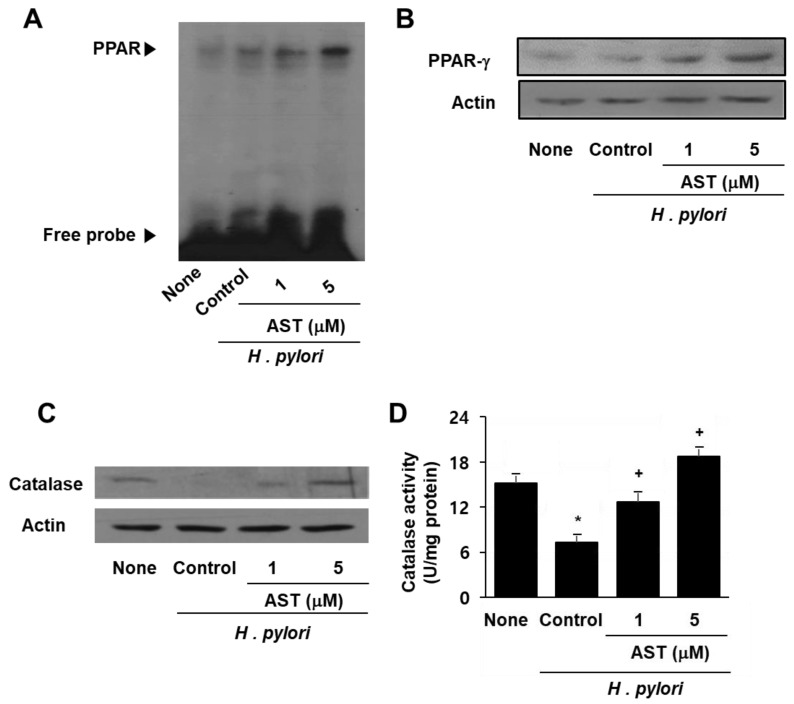
The effect of astaxanthin on the expression and activation of PPAR-γ and catalase in *H. pylori* –infected AGS cells. The cells were pre-treated with the indicated concentrations of astaxanthin for 3 h and then stimulated with *H. pylori* for 1 h. (**A**) DNA binding activity of PPAR was determined by EMSA. (**B**) Protein level of PPAR-γ was determined by Western blot analysis. (**C**) Protein level of catalase was determined by Western blot analysis. (**D**) Catalase activity in the sample was determined by catalase assay kit. * *p* < 0.05 vs. none (cells without any treatment or stimulation); + *p* < 0.05 vs. control (cells with *H. pylori* stimulation alone).

As represented in Figure 4A, the data from the EMSA analysis show that astaxanthin increases the DNA binding activity of PPAR in *H. pylori-*infected cells whereas *H. pylori* itself did not activate PPAR (Figure 4A). Western blot analysis revealed a marked increase in PPAR-γ resulting from astaxanthin treatment in a dose-dependent manner (Figure 4B). Furthermore, astaxanthin restored the expression and activity of catalase in AGS cells, which had been decreased as a result of *H. pylori* infection (Figure 4C,D). These results suggest that the inhibitory effect of astaxanthin on *H. pylori*-induced ROS production and IL-8 expression stems from PPAR-γ induction and activation as well as increased expression and activity of the PPAR-γ target enzyme catalase. Antioxidant enzyme catalase may scavenge and reduce the H_2_O_2_ that inhibits ROS-mediated activation of NF-κB and IL-8 gene expression in *H. pylori*-infected cells.

### 3.5. PPAR-γ Antagonist Abolishes the Inhibitory Effect of Astaxanthin on Elevated ROS Levels and IL-8 Gene Expression in H. pylori—Infected AGS Cells

**Figure 5:**
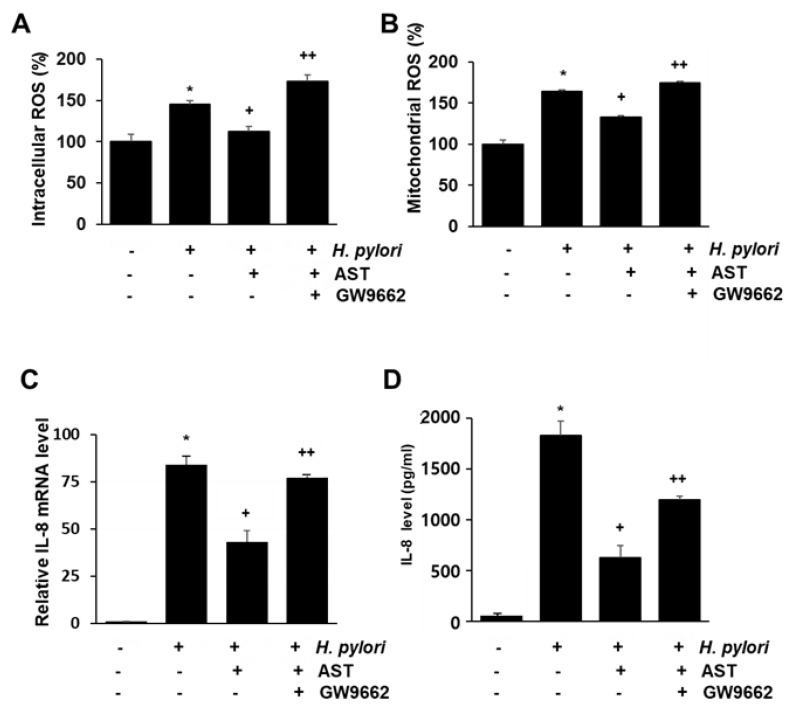
The effect of GW9662 on ROS levels and IL-8 expression in *H. pylori*—infected AGS cells treated vs not treated with astaxanthin. The cells were treated with astaxanthin (5 μM) for 3 h without, or in combination with, the PPAR-γ antagonist GW9662 (5 μM), and then stimulated with *H. pylori* for 1 h (for intracellular and mitochondrial ROS levels), 4 h (for IL-8 mRNA level), and 24 h (for IL-8 protein level in the medium). (**A**) A plot of the relative intracellular ROS levels measured by DCF-DA fluorescence. (**B**) A plot of the relative mitochondrial ROS level measured by MitoSOX fluorescence. (**C**) A plot of the relative mRNA expression of IL-8 determined by real-time PCR analysis. (**D**) A plot of the concentration of IL-8 in the media determined by ELISA. * *p* < 0.05 vs. cells without any treatment or stimulation; + *p* < 0.05 vs. cells with *H. pylori* stimulation alone; ++ *p* < 0.05 vs. cells with astaxanthin treatment and *H. pylori* stimulation. “−“ means without treatment or stimulation. “+” means with treatment or stimulation.

To determine if PPAR-γ is the mediator of the inhibitory action of astaxanthin on *H. pylori*-stimulated AGS cells, the impact of the PPAR-γ antagonist GW9662 was determined. Accordingly, the intracellular and mitochondrial ROS and the IL-8 mRNA and protein were measured for native and *H. pylori*-infected AGS cells as a function of pretreatment with 5 μM astaxanthin alone, or in combination with 5 μM GW9662 (see Figure 5A–D). The inhibitory effect of astaxanthin on *H. pylori*-induced increase in intracellular and mitochondrial ROS levels (Figure 5A,B) and IL-8 gene expression (Figure 5C,D) is suppressed by co-treatment of GW9662. These results suggest that PPAR-γ mediates the anti-inflammatory action of astaxanthin in *H. pylori*-infected cell by reducing intracellular and mitochondrial ROS levels and IL-8 gene expression.

## 4. Discussion

*H. pylori* infection induces the production of inflammatory cytokines leading to gastric inflammation. It is well known that an increased level of IL-8 is associated with *H. pylori* infection [33]. Whole genome profiling of *H. pylori*-infected gastric epithelial cells revealed that the IL-8 gene is the one that is most upregulated [34]. The results of this study are in line with previous studies, which have shown that stimulation of AGS cells with *H. pylori* results in increased IL-8 levels. 

One of the well-known mechanisms of IL-8 induction by *H. pylori* involves the production of ROS [35]. Many studies have identified ROS as signaling mediators in *H. pylori*-stimulated IL-8 induction. *H. pylori* is thought to activate NADPH oxidase, resulting in overproduction of ROS. The present study shows that *H. pylori* stimulation increased the activity of NADPH oxidase in AGS cells. The NADPH oxidase inhibitor apocynin blocked *H. pylori*-induced ROS production and IL-8 gene expression, which demonstrates that NADPH oxidase mediates mitochondrial ROS-stimulated IL-8 gene expression. Because astaxanthin has no effect on NADPH oxidase, the inhibitory effect of astaxanthin on IL-8 gene expression does not involve the NADPH oxidase. 

Another major source of ROS in cells is mitochondria. Mitochondria produce superoxide anions as electrons leak from the mitochondrial electron transport chain. Under normal physiological conditions, ROS generated by mitochondria are removed by antioxidant enzymes. However, when the mitochondria are damaged, as occurs under pathological conditions, mitochondrial ROS production rises to a level that overruns the antioxidant defense system [18,36]. Mitochondrial dysfunction is often observed in oxidative stress-related diseases such as atherosclerosis and age-related degenerative diseases, where oxidative damage impairs mitochondrial function and propagates mitochondrial ROS production [16,36,37,38]. That excess levels of mitochondrial ROS, which act as signaling molecules, aggravate inflammatory response is well known. However, the involvement of mitochondrial dysfunction in *H. pylori*-induced gastric inflammation had not been previously studied. Our findings show that *H. pylori* infection decreases MMP and ATP levels in the cells, indicating a disturbance in mitochondrial function. Furthermore, our results show that *H. pylori* infection induces mitochondrial dysfunction by increasing NADPH oxidase-mediated ROS production.

Astaxanthin is well known for its antioxidant capacity and previous studies have shown that it can mitigate inflammation through its antioxidative action. Moreover, several studies revealed that astaxanthin has a protective effect on the mitochondria. Astaxanthin prevented the loss of MMP and a drop of mitochondrial respiration rate in in vitro and in vivo models of cardiac injury as well as in human neuroblastoma cells [39,40,41,42]. We observed that astaxanthin protects against *H. pylori-*induced mitochondrial dysfunction in AGS cells, as indicated by its restoration of MMP and ATP level.

*H. pylori* infection generates excess amounts of ROS, which in turn activate NF-κB which leads to the expression of IL-8 [10,11]. Our study revealed that increased activation of NF-κB occurs in AGS cells infected by *H. pylori* and showed that NF-κB activation by *H. pylori* is suppressed by astaxanthin.

PPAR-γ ligands and agonists can help attenuate the inflammatory response by activating the synthesis of antioxidant enzymes, which in turn scavenge oxidative radicals. Inoue et al. [32] reported that astaxanthin acts as a PPAR-γ agonist in oxidative stress-related conditions in macrophages. In the present study, astaxanthin was found to increase PPAR activation and PPAR-γ expression in *H. pylori*-stimulated AGS cells. Astaxanthin further increased the expression and activity of catalase in AGS cells. Furthermore, we showed that the inhibitory effect of astaxanthin on the *H. pylori*-induced increase in intracellular and mitochondrial ROS levels and IL-8 gene expression, is suppressed by treatment with the PPAR-γ antagonist GW9662. Therefore, the inhibitory effect of astaxanthin on *H. pylori*-induced, ROS-stimulated IL-8 expression may be mediated by the antioxidant capacity of astaxanthin and its effect on PPAR-γ.

Taken together, the present study supports the proposal that astaxanthin inhibits *H. pylori*-induced IL-8 expression by alleviating oxidative stress from ROS through upregulation of catalase, thus preventing oxidative stress-induced mitochondrial dysfunction. Preventing mitochondrial dysfunction in turn reduces the production of mitochondrial ROS and blocks the activation of inflammatory mediators such as NF-κB.

In the present study, we showed that *H. pylori* promoted a slight increase of PPAR-γ activation. Several studies have revealed that PPAR-γ is upregulated by *H. pylori* in gastric epithelial cells and gastric carcinoma cells [43,44,45]. However, the studies indicate that the PPAR-γ level is elevated through an instantaneous defense mechanism orchestrated by the gastric mucosa, and that the change is comparatively insignificant relative to that associated with PPAR-γ activation triggered by PPAR-γ ligands or agonists [44,45]. Indeed, astaxanthin had a stronger effect on PPAR-γ activation. Moreover, cells treated with astaxanthin had significantly higher catalase activity compared to those subjected to *H. pylori* infection alone. 

The concentrations of astaxanthin and time points for determining ROS, NF-κB, and IL-8 used in the present study were adapted from the previous studies [46,47,48,49,50]. Solomonov et al. [46] showed that the anti-inflammatory effect of 1 μM astaxanthin was similar to that of 2 μM astaxantin in in vitro and in vivo mouse model of peritonitis. In the present study, we used 1 and 5 μM astaxanthin to determine the inhibitory effect of astaxanthin on IL-8 expression in *H. pylori*-infected gastric epithelial cells. Further studies for determining the inflammatory effect of 2.5 μM astaxanthin on *H. pylori*-induced IL-8 expression may provide a dose-dependent anti-inflammatory effect of astaxanthin. Previous studies showed that *H. pylori* induced ROS production and NF-κB activation at 1 h [47,48,49], IL-8 mRNA expression at 4 h [50] and IL-8 protein expression at 24 h [48,49] in gastric epithelial AGS cells. Thus, in the present study, following 1 h of incubation, the ROS levels and NF-κB DNA binding activity were determined. Following a 4 h incubation period, the IL-8 mRNA expression was measured. And following 24 h, the IL-8 level in the medium was assessed. 

In regard to culture condition, *H. pylori* was added to the AGS cells and cultured at 95% air and 5% CO_2_ in the preset study. Park et al. [51] reported that *H. pylori* is a capnophilic aerobe whose growth is promoted in the presence of higher oxygen content (20%) when the concentration of CO_2_ is also high (10%). Therefore, it is necessary to perform the experiment using the culture conditions close to those in stomach (gastric gas contains 5–9% CO_2_ and 15–19% O_2_) for further study. 

In the present study, we pretreated astaxanthin before *H. pylori* infection. It mimics the infection of an individual who habitually ingests astaxanthin from natural or supplemental sources. The present study may demonstrate a certain degree of protection from the effects of *H. pylori* infection. Reversing the sequence of treatment (infection with *H. pylori* before astaxanthin is added to the growth medium) would provide indications whether astaxanthin treatment is helpful in ameliorating gastric inflammation in patients with pre-existing *H. pylori* infection. According to several in vivo studies that investigated the effect of astaxanthin on oxidative stress-inducing stimuli [52,53], both pre-treatment and post-treatment with astaxanthin showed significant reduction in ROS level and oxidative stress markers, as well as significant restoration of antioxidant enzymes involving catalase, superoxide dismutase, glutathione peroxidase, and glutathione. There were no noticeable differences between the pre-treatment and post-treatment group. In addition, though the experimental conditions are quite different, astaxanthin did show significant reduction in bacterial load and inflammation when post-treated to in vivo models inoculated with *H. pylori* [54,55]. To address whether astaxanthin is effective in reducing oxidative stress, mitochondrial dysfunction, and inflammation of patients with pre-existing *H. pylori* infection, the time of treatment (i.e., pre- vs. post-treatment) should be considered in more detail in future in vitro and in vivo works. 

The bioavailability of carotenoids, which are very lipophilic compounds, is low. It varies widely, from less than 10% from raw uncooked vegetables to more than 50% in oily solutions or in synthetic commercial formulations [56]. Wang et al. [55] demonstrated that oral astaxanthin was absorbed and incorporated into mouse gastric mucosa. Using Caco cells, Yang et al. [57] revealed that cellular uptake of astaxanthin was significant starting from 3 h, and its absorption continued to increase until 24 h. Another study by Mimoun-Benarroch et al. [58] showed that astaxanthin from the microalgae *Haematococcus pluvialis*, whose source is identical to that used in the present study, is absorbed by up to 50% after 3 h, and that its absorption did not significantly change by 6 h in Caco cells. Considering that the source of astaxanthin used in the present study is the algae *Haematococcus pluvialis* and that astaxanthin exposure time was longer than 3 h, we infer that a considerable amount of astaxanthin might be absorbed by AGS cells. It is essential to determine the amount of astaxanthin after treatment to the cells in the present system for future study.

The results show that astaxanthin inhibits *H. pylori*-induced mitochondrial dysfunction and ROS-mediated IL-8 expression by activating PPAR-γ and catalase activity in gastric epithelial cells. Inhibition of mitochondrial dysfunction and IL-8 expression by astaxanthin could potentially be exploited to prevent or delay oxidative stress-associated gastric inflammation resulting from *H. pylori* infection.

## 5. Conclusions

This study supports the preventive effect and underlying molecular mechanism of astaxanthin against *H. pylori*-induced gastric inflammation. Astaxanthin activates PPAR-γ and its downstream target gene catalase, thereby reducing ROS and preventing mitochondrial dysfunction. Through this molecular mechanism, astaxanthin significantly suppresses pro-inflammatory cytokine IL-8 gene expression. Therefore, astaxanthin is effective in mitigating oxidative stress and the inflammatory response caused by *H. pylori* infection.
